# Synchronous slowing down in coupled logistic maps via random network topology

**DOI:** 10.1038/srep23448

**Published:** 2016-03-29

**Authors:** Sheng-Jun Wang, Ru-Hai Du, Tao Jin, Xing-Sen Wu, Shi-Xian Qu

**Affiliations:** 1 Institute of Theoretical & Computational Physics, Shaanxi Normal University, Xi’an 710062, China; 2 School of Physics and Information Technology, Shaanxi Normal University, Xi’an 710062, China

## Abstract

The speed and paths of synchronization play a key role in the function of a system, which has not received enough attention up to now. In this work, we study the synchronization process of coupled logistic maps that reveals the common features of low-dimensional dissipative systems. A slowing down of synchronization process is observed, which is a novel phenomenon. The result shows that there are two typical kinds of transient process before the system reaches complete synchronization, which is demonstrated by both the coupled multiple-period maps and the coupled multiple-band chaotic maps. When the coupling is weak, the evolution of the system is governed mainly by the local dynamic, i.e., the node states are attracted by the stable orbits or chaotic attractors of the single map and evolve toward the synchronized orbit in a less coherent way. When the coupling is strong, the node states evolve in a high coherent way toward the stable orbit on the synchronized manifold, where the collective dynamics dominates the evolution. In a mediate coupling strength, the interplay between the two paths is responsible for the slowing down. The existence of different synchronization paths is also proven by the finite-time Lyapunov exponent and its distribution.

Synchronization is a universal phenomenon in physical, biological, chemical, and social systems^1^,^2^,^3^,^4^,^5^,^6^,^7^,^8^,^9^, and also plays an important role in many systems such as secure communication^10^, coupled lasers^11^,^12^, and neuronal networks^13^,^14^,^15^, and so on. During the past decades, there has been a surge of interest in the study of synchronization phenomena in coupled oscillators and coupled map lattices^7^,^16^,^17^,^18^,^19^,^20^,^21^,^22^. However, vast majority of the existing achievements mainly focus on the condition of the onset of synchronization and the stability of synchronized states^23^, little attention turns to the dynamics of the synchronizing process and the speed of synchronization^24^ given that a network synchronizes in principle. While in realistic systems, it equally matters how fast the units synchronize or whether the network interactions fail to coordinate the units’ dynamics on time scales relevant to the system’s function^24^,^25^,^26^,^27^,^28^. One typical example is that in neuroscience the above issue is related to the speed of the visual processing or olfactory discrimination^29^,^30^.

Along this line, the study of synchronization process has attracted some attention^24^,^31^,^32^. In a recent work^24^, the effect of network topology on the synchronization time was analyzed. The synchronization time monotonically decreases with the topological randomness when the networks have fixed in-degree. Moreover, the synchronization time of networks with fixed average path length non-monotonically depends on the topological randomness. At the boundary of synchronization parameter region, the critical slowing down of convergence was obtained^33^. For logistic maps on a chain, it was found that the average synchronization time non-monotonically changed with the coupling parameter, but this phenomenon only took place when the coupled map lattice size was very small (the size N = 5)^31^. Actually, the question of synchronization speed were far from being understood and currently under active investigation as well^24^.

In this paper, we study the synchronizing process and the synchronization time of coupled logistic maps on random networks. The logistic map is widespread concerned^34^,^35^,^36^,^37^,^38^ since it has become a prototype for studying chaos theory and reflects common features of low-dimensional chaotic dissipative systems. We present a novel relation between synchronization time and coupling strength, the synchronization speed slows down at a mediate coupling strength, and the mechanism of this phenomenon roots in the transition of synchronization paths. These results may help us to deeply understand the synchronization of coupled nonlinear elements.

The dynamics of coupled maps are described by the following equation,


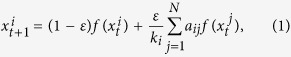


where *f*(*x*) denotes the map function on the single node in the network, *ε* (0 < *ε *< 1) is the coupling strength, and 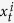 stands for the state of the *i*-th node at the *t*-th step. In this work, the single map *f*(*x*) takes the form of the logistic map *f* (*x*) = *μx*(1 − *x*), with the control parameter *μ* ∈ [0, 4], and the dynamical variable *x* ∈ [0, 1]. {*a*_*ij*_} is the adjacency m*a*trix, *a*_*ij *_= 1 indicates that node *i* and node *j* are connected. If *a*_*ij*_ = *a*_*ji*_ = 0, there is no coupling between them. 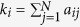 indicates the connection degree of the *i*-th node.

The logistic maps are coupled through a random network topology. To build the random network, we randomly select a pair of nodes and establish a link between them if they are not connected by an existed link. Repeat this procedure up to the number of links is *N*(*N* − 1)*p/*2, where *N* is the network size and *p* is the connection probability of each pair of nodes^39^. The average degree of nodes is *Np*. In the present work, the system consists of *N* = 1000 nodes, and the connection probability *p* = 0.02.

For coupled logistic maps on random networks, there is in general a threshold of coupling strength, above which the system may approach a stable synchronization state after a transient process of finite time period. In the current work, the coupling strength is set above this threshold so that the maps in the network can reach synchronization from arbitrary states. In the simulation, the initial values 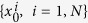 of the maps are assigned to a set of random numbers uniformly distributing in [0, 1]. The time step at which the system reaches synchronization is called the synchronization time and denoted by *t*_s_.

Synchronization order parameter *R* is used to determine whether the system achieves synchronization, which is defined by the following equation,


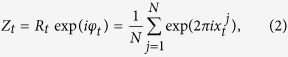


where *R*_*t*_ is the amplitude of *Z*_*t*_ at *t*-th step, *φ*_*t*_ is the corresponding phase. When *R*_*t *_= 1, all of the nodes have the same state, 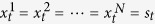, i.e., the system is synchronized. Here, *s*_*t*_ is the synchronized orbit, which is the same to that of the single map. In order to describe how far the state of the system is from the complete synchronization state during evolution, the deviation of the synchronization order parameter *R* from its maximum value 1 is employed, which is defined by


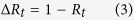


where the index *t* represents the time step.

## Results

**Figure 1:**
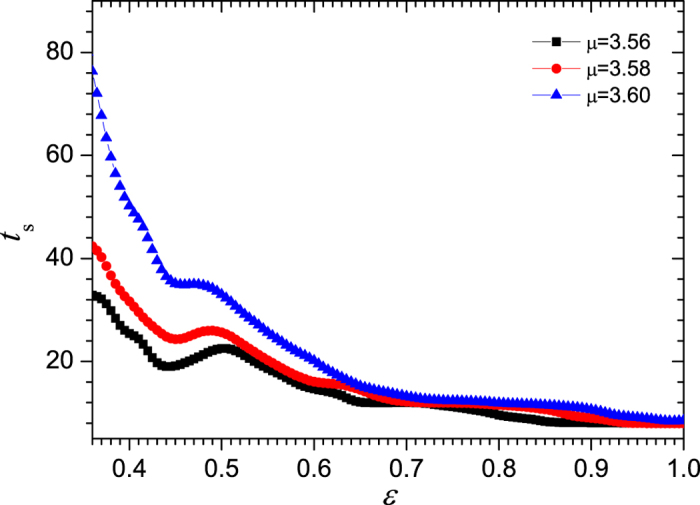
The synchronization time *t*_*s*_ versus the coupling strength *ε*. Where, the ensemble average is done for 10^5^ realizations. The control parameters of the logistic maps are respectively *μ* = 3.56, 3.58, and 3.60. The size of the network is *N* = 1000, and the average degree is 20. The threshold of synchronization is Δ*R*_*t*_ < 10^−8^.

In the current work, the synchronization time is obtained by the average of *t*_*s*_s over 10^5^ realizations for each coupling strength *ε* ∈ [0.36, 1]. The synchronization criterion is set to Δ*R* ≤ 10^−8^ since we only concern the transient process before the complete synchronization. One might generally expect that, given all the other parameters, *t*_*s*_ is a decreasing function of the coupling strength since it seems reasonable that larger coupling strength tends to accelerate the synchronization. However, the simulation result in this work shows that the synchronization time can not be described by a monotonic function of the coupling strength, as shown in Fig. 1, in which the time series of the synchronization time for three different control parameters *μ* of the logistic map are drawn. The single map is in period-8 state when *μ* = 3.56. While when *μ* = 3.58 and 3.60, the single map is in the 4-band and 2-band chaotic state, respectively. Here *n*-band means that the chaotic attractor consists of *n* separated ranges in the phase space. In each of the plots, there is a peak around the middle of the coupling strength. The synchronization speed is slowed down at a mediate coupling strength.

**Figure 2:**
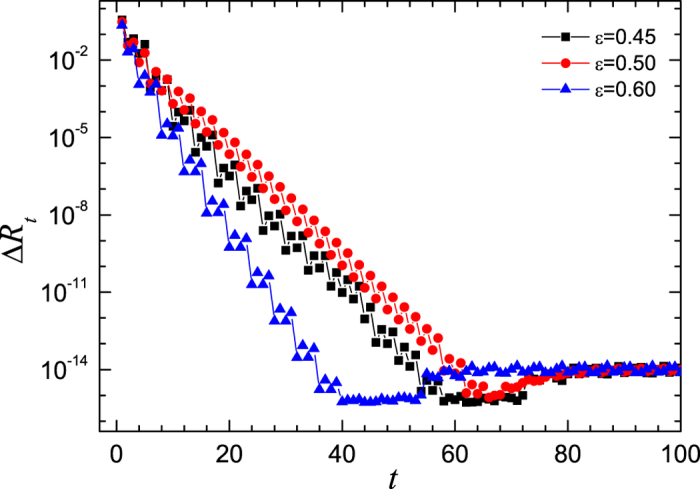
The time series of Δ*R* for three different coupling strengths. Where, the ensemble average is done for 10^5^ realizations. The squares represent the case for *ε* = 0.45, the circles represents that for *ε* = 0.50, and the triangles for *ε* = 0.60. The control parameter of the logistic map is *μ* = 3.56.

To understand the slowing down of the synchronization speed, a detail analysis of the synchronization processes is presented in the follows. First of all, the network of period-8 maps is considered. The evolutions of the system dynamics are simulated under three different values of coupling strength, i.e., *ε* = 0.45 (at the left of the peak), *ε* = 0.50 (around the peak), and *ε* = 0.60 (at the right of the peak). The time series of the deviation Δ*R*_*t*_ are plotted in Fig. 2, in which the values of Δ*R*_*t*_ are obtained by the ensemble average over 10^5^ realizations. One can see that the deviation from the synchronized states decay exponentially before the complete synchronization. The slopes of the semi-log plots of Δ*R*_*t*_ versus *t* in Fig. 2 describe the decay rates of the deviation (where, the stair like appearance is due to the period-8 structure of the attractor). The slopes for the cases of *ε* = 0.45, *ε* = 0.50 and *ε* = 0.60 are respectively −0.243 ± 0.005, −0.213 ± 0.003 and −0.370 ± 0.008. As expected by the intuition, the decay rate of Δ*R* in the system with strong coupling is faster than that in the system with weak coupling. However, for the system with *ε* = 0.50, the decay rate is smaller than those in both the weak and the strong coupling strengths, which indicates that the mediate coupled system has different dynamics for converging into the stable synchronous states. This is a novel phenomenon.

**Figure 3:**
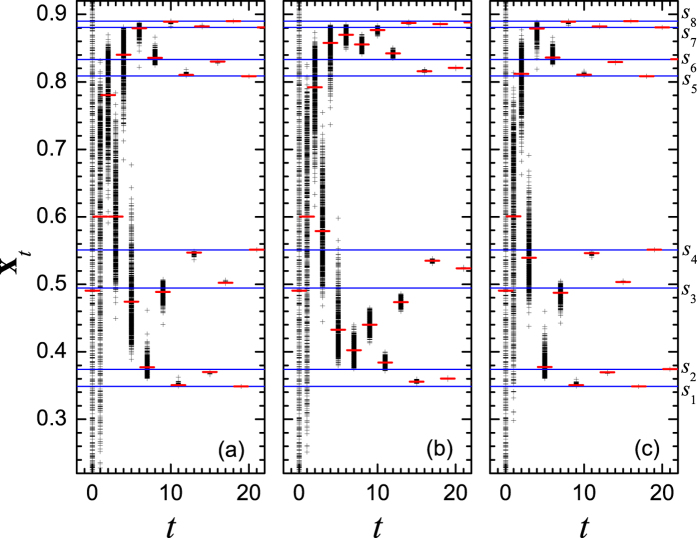
The time series of node states *x*_*t*_ for three different coupling strengths: (**a**) *ε* = 0.45, (**b**) *ε* = 0.50, and (**c**) *ε* = 0.60. The plus (+) indicates the state on each node, the minus (−) indicates the average value of all node states. The lines indicates the trajectories of P-8 orbit, marked by *s*_1_, *s*_2_, ..., and 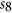, respectively. The control parameter of the single map is *μ* = 3.56.

**Figure 4:**
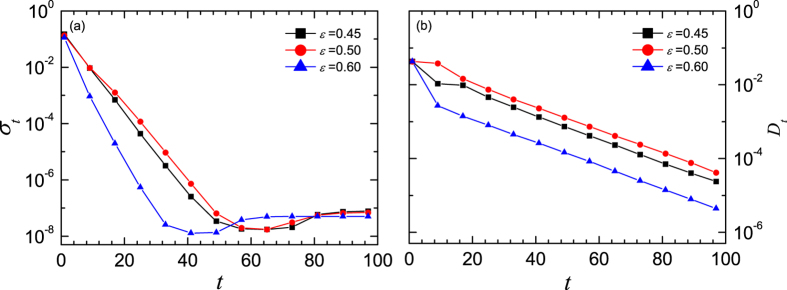
The time series of the standard deviation of node states (**a**) and the distance of the mean state to the stable orbit (**b**). Where, the ensemble average is done for 10^5^ realizations. For clearness, the data points are drawn at every 8-step, considering the P-8 cycle. The squares represent the case of *ε* = 0.45, the circles represent that of *ε* = 0.50, and the triangles are for *ε* = 0.60. The control parameter of the single map is *μ* = 3.56.

To explain why there appears the non-monotonic relation between the average synchronization time *t*_*s*_ and the coupling strength *ε*, the typical time series of the states on nodes 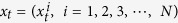 for three values of coupling strength are shown in Fig. 3, in which the eight states of the period-8 (P-8) cycle are illustrated by the horizontal lines and denoted by *s*_1_, *s*_2_, …, and *s*_8_, respectively. For *ε* = 0.45, Fig. 3(a) shows that the values of states are firstly attracted into the neighborhood of the stable orbit of the single map, and then tend to the synchronized orbit of the P-8 cycle almost individually as the time goes, exhibiting a less correlated evolution. In contrast, the system undergoes a completely different dynamic process when the coupling strength *ε* = 0.60. In this case, as shown in Fig. 3(c), the distribution of the trajectories shrinks into a narrow range very soon, which implies that the states of nodes evolve in a more coherent way, or say, they tend to the synchronized orbit in a collective way. Therefore it is much easier to reach the synchronized P-8 cycle. To verify this picture, the standard deviation of node states and the distance of the mean value of the node states to the stable orbit at time *t* are respectively defined by


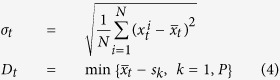


where *P* is the period and 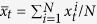 is the mean state. The evolution of them for three *ε* values are plotted in Fig. 4, where the data points are drawn at every 8-step for clearness, considering the P-8 structure of the attractor. Obviously, both *σ*_*t*_ and *D*_*t*_ for *ε* = 0.45 are larger than those for *ε* = 0.60. On the average, *σ*_*t*_ of the former is 19 times larger than that of the latter, and the corresponding ratio for *D*_*t*_ is 2.5. While for *ε* = 0.50, as shown in Fig. 3(b), the individual states do not evolve along the stable orbit of either the single map or the synchronous manifold, but spend a pretty longer time to fluctuate violently in the phase space, especially in the regions confined by (*s*_2_, *s*_3_) and (*s*_6_, *s*_7_). Here, *σ*_*t*_ is approximately 3 and 62 times larger than those for *ε* = 0.45 and *ε* = 0.60, respectively. The corresponding ratios for *D*_*t*_ are approximately 3 and 5. Thus, the synchronous slowing down appears in the mediate coupling strengths. To verify whether the above mentioned phenomenon is due to the existence of the cluster states, careful analysis on the spatiotemporal amplitude plots for different initial conditions were carried out. The result shows that there is no cluster state appearing before the complete synchronization. Thus, there might be other mechanism than the multiple cluster states.

Actually, the phenomenon might be easily understood by considering the basic feature of eq. (1), which shows that the dynamics of the coupled systems is governed by the competition between the local dynamics and the coupling term. In the case of weak coupling, the local dynamics dominates the evolution of the system and the interaction is not strong enough to make the states of nodes evolve in a high coherent way. Thus the trajectories of the maps oscillate around the stable P-8 orbit of the single map except the very early age of evolution, and tend to synchronization almost individually. While in strong coupling, since the contribution to the system dynamics from the interaction term is superior to that from the local ones, the collective dynamics dominates the evolution of the system, making the maps to oscillate in a more coherent way around the synchronized orbit. It is clearly shown in Fig. 3(c), in which the maps tend to almost the same state before they converge into the stable P-8 orbit. In a mediate coupling strength, the contribution of the interaction term is comparable to that due to the local dynamics. It is not strong enough to force the node states 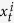 evolve in a high coherence way, but is sufficient to prevent the states from converging to the stable orbit individually. Thus, at least in the early period, the trajectories can not evolve around the stable orbit, but fall in the regions between the periodic points, showing strong oscillation (as shown by Fig. 3(b)). Then, one may conclude that the competition or interplay between the two types of dynamics results in the crossover of the synchronization time, and thus a peak is observed in the *t*_*s*_ ~ *ε* curve marked by solid squares in Fig. 1, i.e., the case for *ε* = 0.50. Moreover, the multiple period and multiple band structure of the attractor of the single map might have some contribution to the slowing down of synchronization, which will be discussed in the later part of the paper.

Since we are interested in the synchronizing process, the finite time Lyapunov exponent (FTLE) and its distribution might be employed to characterize and explain the synchronous slowing down. The FTLE describes the amount of stretching and contracting around a trajectory 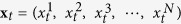 over the finite time interval [*t*, *t* + *τ*]. We consider a neighbor trajectory y_0_ around x_*t*_ with any small displacement *d*. After evolving for a finite time *τ* by eq. (1), the two neighbor trajectories move to x and y, respectively. Then the FTLE is calculated by


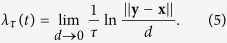


In our calculation, the displacement is set to *d* = 10^−8^, and 10^5^ sets of random initial values distributed uniformly in [0, 1] are chosen for ensemble average and the corresponding distribution of the FTLEs is also calculated. The finite time duration *τ* is carefully selected for different coupling strengths since the improper choice of them may induce wrong result^40^.

**Figure 5:**
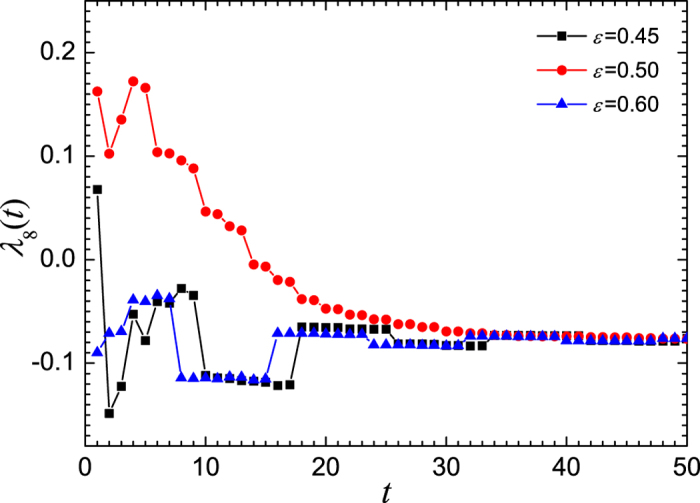
The time series of finite time Lyapunov exponent *λ*_8_(*t*) when *μ* = 3.56. Where, the ensemble average is done for 10^5^ realizations. The squares represent the case of *ε* = 0.45, the circles represent that of *ε* = 0.50, and the triangles are for *ε* = 0.60.

The variation of the FTLE *λ*_8_(*t*) when *μ* = 3.56 is shown in Fig. 5. Obviously, in a considerable long time period, the FTLE for *ε* = 0.50 is much larger than the maximum Lyapunov exponent *λ*_*max*_ = −0.0771445 of the coupled system (also the Lyapunov exponent of the single map) and even greater than zero in the early age of the evolution. This may be explained by Fig. 3(b), in which the state of nodes distributes in the regions between pairs of adjacent stable periodic points. In each of these regions, there is an unstable fixed point which makes the states of the coupled system jump between different basins of the fixed points and is thus responsible for the larger FTLEs. While for *ε* = 0.45 and 0.60, the FTLEs oscillate around *λ*_max_ with an amplitude much smaller than the deviation of FTLEs when *ε* = 0.50. For these two cases, Fig. 3(a,c) reveal that the node states evolve in the vicinity of the stable orbit. Therefore, we may conclude that the larger FTLE is an intrinsic dynamical reason for the slowing down of the synchronization in a mediate coupling strength.

**Figure 6:**
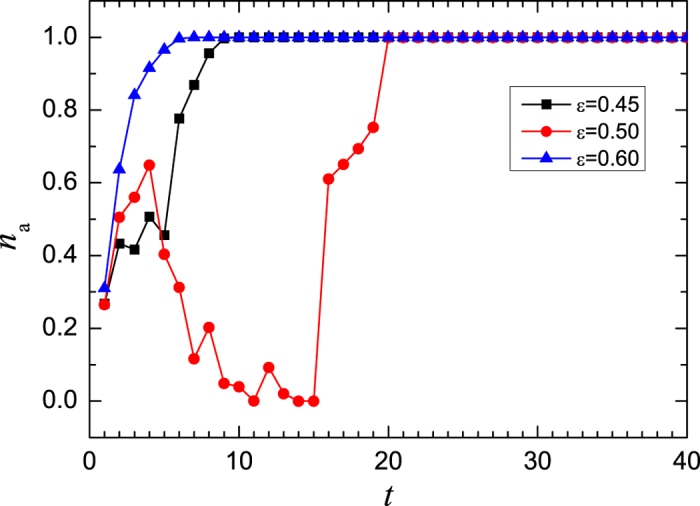
The evolution of the fraction of maps whose trajectories fall in the chaotic attractor. Where, the ensemble average is done for 10^5^ realizations. The parameter of the logistic map is *μ* = 3.58.

Now, we turn to discuss the case when *μ* = 3.58 where the dynamics of the single map is in chaotic state and the synchronization time also exhibits a non-monotonic dependence on the coupling strength. The time series of Δ*R*_*t*_ shows the similar behavior. Here the chaotic attractor consists of four separated regions in the phase space, which attracts the trajectories outside them. To illustrate the attraction of the chaotic attractor, we calculate the number fraction of the maps whose state falls in the four chaotic bands, which is denoted by *n*_*a*_. Figure 6 shows the evolution of *n*_*a*_ for three coupling strengths *ε* = 0.45, 0.5 and 0.6 which represents the weak, mediate and strong couplings, respectively. For the weak coupling, the value of *n*_*a*_ increases to about 0.4 very soon and stays there for several steps. Then the state of more maps is attracted into chaotic bands, and *n*_*a*_ increases to 1.0 afterward. For the strong coupling, the state of maps enters chaotic bands quickly. However, at the mediate coupling strength, *n*_*a*_ goes down to very small value after a very short increasing, and then increases slowly comparing with the previous two cases. It spent much longer time to have the state of all the maps falling in the attractor. Thus, the variation of *n*_*a*_ can also somehow demonstrate the synchronous slowing down.

**Figure 7:**
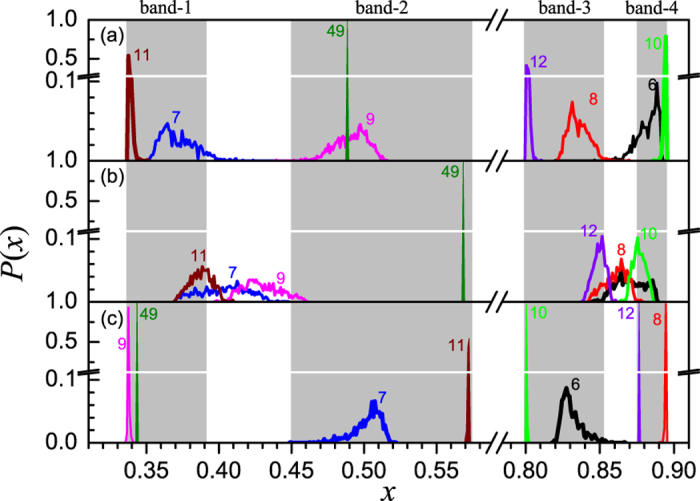
The distribution of node states for a typical initial condition when *μ* = 3.58. In (**a**–**c**) the coupling strengths are *ε* = 0.45, 0.50 and 0.60, respectively. Gray background represents the chaotic bands of the logistic map.

To display the evolution of the system in more detail, shown in Fig. 7 are the typical distributions of node states. In both the weak and strong couplings, i.e., Fig. 7(a,c), almost all states fall in the chaotic bands. One may find that the peaks in the latter case are sharper than those in the former case, especially after the 6-th time step, which implies that the system evolves in a weak coherent way in weak coupling, but in a high coherent way in strong coupling. We may call them respectively the weak and high coherent transients of synchronization, respectively. While when *ε* = 0.50, a very different situation appears, where a major portion of the node states falls in two regions out side the chaotic bands in the phase space, one is between band-1 and band-2 and the other between band-3 and band-4. Furthermore, the states distribute over more broaden ranges than in previous two cases. In this mediate interaction strength, since the contributions of the single map and the coupling term are comparable, the system’s evolution can obey neither the local dynamics nor the collective dynamics. Thus, the states can hardly be attracted into the chaotic bands, especially in the early period of the evolution. Therefore, we may conclude that the competition between the two tendencies make the system spend a longer time to approach the synchronized state. We have to point out that the distributions of state for all the three coupling strengths collapse into one curve with the height of peaks equal to 1 after a pretty long time, e.g., the 49-th time step in the figure, where the system reaches the synchronized state.

**Figure 8:**
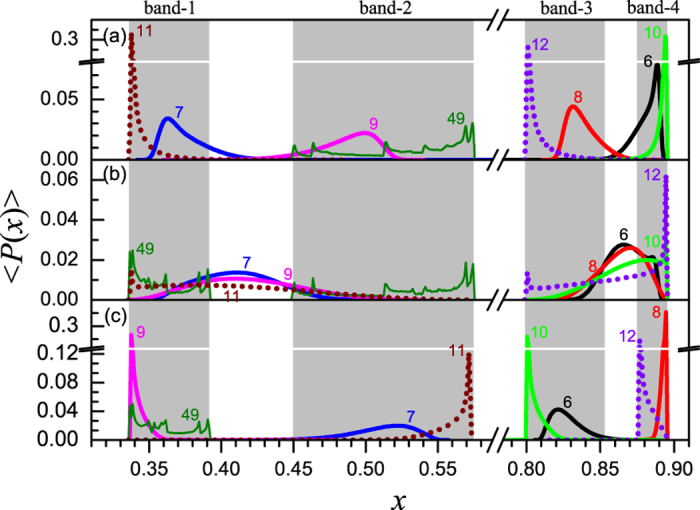
The ensemble average of the distribution of node states when *μ* = 3.58. Where, the ensemble average is done for 10^5^ realizations. In (**a**–**c**) the coupling strengths are *ε* = 0.45, 0.50 and 0.60, respectively. Gray background represents the chaotic bands of the logistic map.

We would also like to present the ensemble average of the state distributions, which are plotted in Fig. 8. Here, in the early time period, the distribution curves become smooth and broaden, because different state distributions corresponding to a large number of initial conditions are involved in the statistics and the ensemble average smooths out the fluctuations due to different types of initial conditions. When *ε* = 0.45, a major portion of states falls within a single band at each time step (see Fig. 8(a)). While when *ε* = 0.60, almost all states fall in a single band at each time step and the peaks become sharper than in previous case (see Fig. 8(c)). As for *ε* = 0.50, the distribution curves extend across two adjacent chaotic bands, and a major portion of states falls outside the bands and the peak are positioned in the middle of the gaps between them. The cause is similar to that in the P-8 case, i.e., the interplay between the local dynamics and the coupling term makes the trajectories of nodes be attracted by neither the attractor of single map, nor the attractor on the synchronized manifold. Another main difference is that, at very late time steps, e.g., the 49-th step, there appear multiple sharp peaks in the distribution curves. It is because that the system reaches almost synchronized state at this moment and the ensemble average includes variety states with different types of initial condition, where each peak is corresponding to the synchronization time for a given type of initial condition.

**Figure 9:**
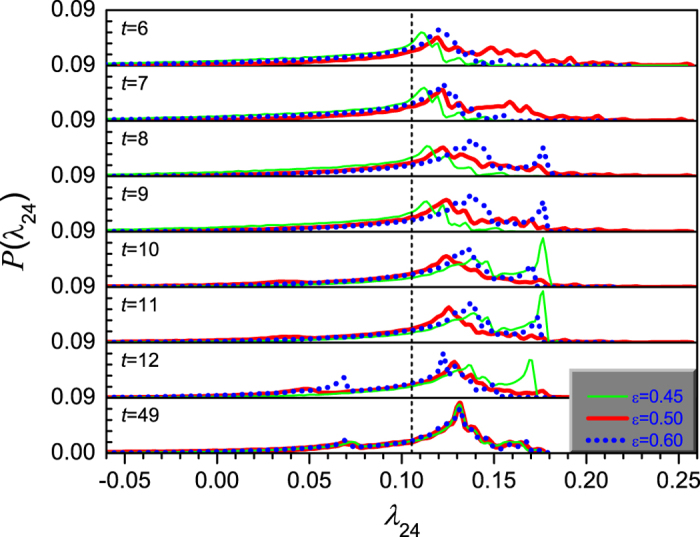
The distribution of finite-time Lyapunov exponent *λ*_24_(*t*) when *μ* = 3.58. Where, the ensemble average is done for 10^5^ realizations. The thin lines denotes the distribution when *ε* = 0.45, the thick lines denotes that when *ε* = 0.50, the dot lines are for *ε* = 0.60, and the dash lines mark the position of the maximum Lyapunov exponents of the system.

Similarly, we will also take a look at the FTLEs. Here, we present in Fig. 9 the ensemble average of the distributions of the FTLEs over 10^5^ realizations. One may observe a very good correspondence with Figs 7 and 8. The distributions of FTLEs for three different coupling strengths *ε* = 0.45, 0.50 and 0.60 show multiple-peak structure, corresponding very well to the multiple peaks in the distributions of node states. Firstly, we focus on the distribution of the FTLEs when *ε* = 0.45, i.e., the thin lines in the figures. During time steps 6–9, there are about 4 distinct peaks that are due to different types of initial conditions. The principal peak is positioned around 0.11 which is a bit larger than the maximum Lyapunov exponent of the system, i.e., *λ*_max_ = 0.105400, where the distribution of node states falls almost inside one chaotic band and the peak position is in the middle of the chaotic band at each time step, as shown in Figs 7(a) and 8(a). During time steps 10 to 12, the right shift of the peaks occurs and also the strongest peak appears in the far right end, which is due to the fact that the node states are positioned near the boundaries of the chaotic bands (see Fig. 8(a)), where the system is very unstable. Secondly, we observe the distribution of the FTLEs when *ε* = 0.60, i.e., the dot lines in the figures. At time steps 6 and 7, it also shows multiple peaks with the mean peak around *λ*_24_ = 0.12 > *λ*_max_. From time step 8 to 11, there also appear the right shift of the peaks, and a much strong sub-peak emerges in the far right end where the peaks of the state distribution are positioned in the vicinity of the boundaries of the chaotic bands (see Figs 7(c) and 8(c)). At step 12, the principal peak shifts back to *λ* = 0.12 since the states of all nodes are distributed within chaotic band-4 (see Fig. 8(c)). Finally, we consider the case of *ε* = 0.50, in which the number of peaks is much more than in the previous two cases and the distribution of FTLEs occupies much larger range along the 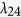-aixs at almost all time steps. After some late time step, e.g., *t* = 49, the three distributions of FTLEs fall in nearly one curve, which implies that the effect on synchronization time and the FTLEs due to the difference of the coupling strengths reduces considerably in very long time of evolution. Therefore, one may say that the difference in the synchronization time due to different coupling strengths emerges mainly at the early period of the evolution. This is the reason why we are interested in the transient process of synchronization.

**Figure 10:**
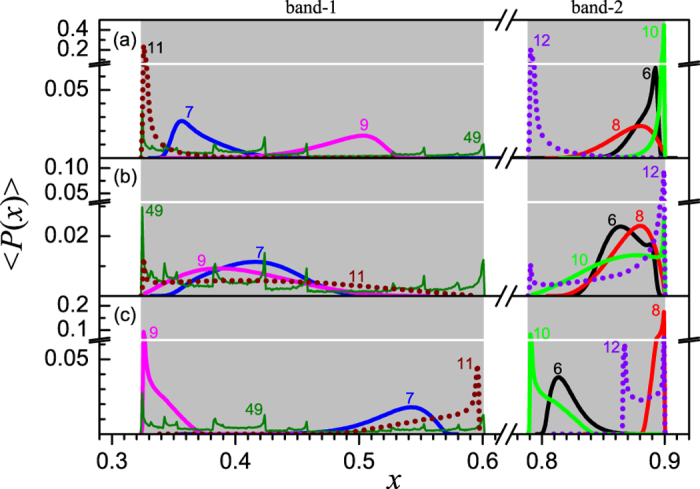
The ensemble average of the distribution of node states when *μ* = 3.60. Where, the ensemble average is done for 10^5^ realizations. The thin lines denote the distribution when *ε* = 0.45, the thick lines denote that when *ε* = 0.50, and the dot lines are for *ε* = 0.60.

**Figure 11:**
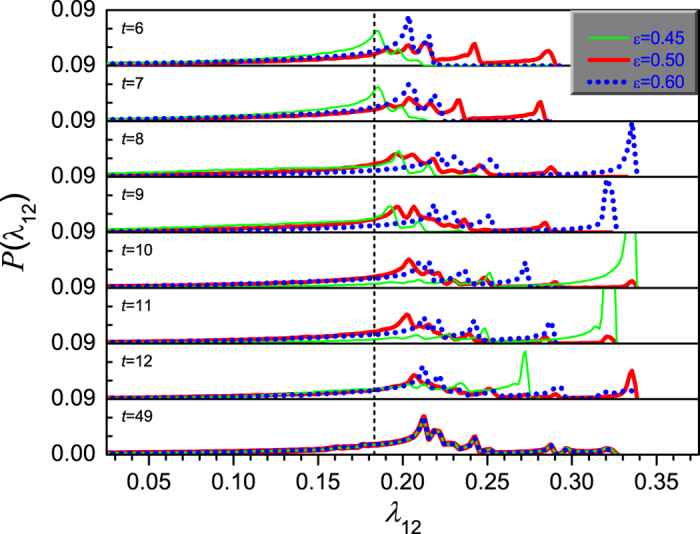
The distribution of finite-time Lyapunov exponent when *μ* = 3.60. Where, the ensemble average is done for 10^5^ realizations. The thin lines denote the distribution when *ε* = 0.45, the thick lines denote that when *ε* = 0.50, the dot lines are for *ε* = 0.60, and the dash lines mark the position of the maximum Lyapunov exponent of the system.

For the coupled maps at 2-band chaotic state when *μ* = 3.60, the distributions of node states and FTLEs are also calculated, which are shown in Figs 10 and 11, respectively. The plots show similar behaviors to those in Figs 8 and 9. However, the main difference is that the coupling strength has no effect on the ranges of the distributions. They are always within one chaotic band at each time step. This implies that the synchronization time in the mediate coupling strength is not necessarily longer than that in the weak and strong coupling cases. Therefore, there is only an indistinct peak in the *t*_s_ ~ *ε* curve around *ε* = 0.50 for *μ* = 3.60 in Fig. 1.

Considering this situation, we may suggest that, besides the competition between the local dynamics and the coupling term, the multiple-period or the multiple-band structure of the single map also contribute to the slowing down of the synchronous process. Actually, the basins of the P-8 fixed points or the chaotic bands are interweaved. A small perturbation may sometimes cause the trajectories jump from one basin of the P-8 fixed points or a chaotic band to another, and thus results in different ability to synchronize. The interaction term in the system of coupled maps provides this perturbation and thus induces the competition among the attractions from different periodic points or chaotic bands. In weak couplings, the system’s behavior is governed mainly by the local dynamics and a small coupling term can not induce frequently violent jumping between different basins. In strong couplings, the system evolves according to the collective dynamics, and the strong coherence make the node states not easy to jump individually and thus also inhibits the competition among different periodic points or chaotic bands even though the contribution of the coupling term is bigger. While in a mediate coupling, the effect of this kind of competition becomes obvious since the interplay between the local dynamics and the collective dynamics is important, and thus enhances the contribution to the slowing down. Hence the synchronization speed depends not only on the coupling strength, but also on the inherent structure of the attractor, especially in the early age of the evolution.

**Figure 12:**
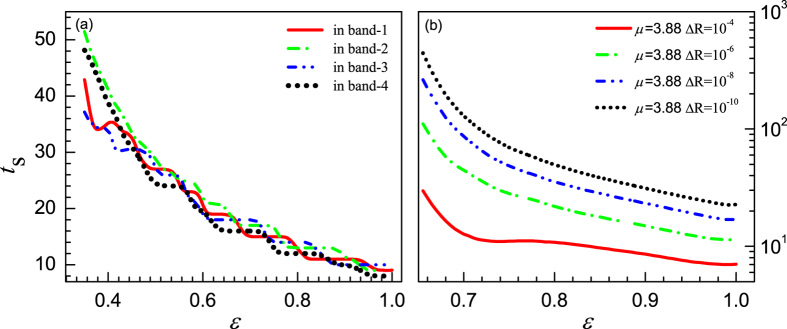
The synchronization times versus *ε*. Where, the ensemble average is done for 10^5^ realizations. (**a**) For *μ* = 3.58 with the initial states randomly distributed in the four chaotic bands, respectively. The threshold of synchronization is Δ*R*_*t*_ < 10^−8^. (**b**) For *μ* = 3.88 with the initial states randomly selected in (0, 1). Different thresholds of synchronization are used.

To further prove this, we re-calculate the synchronization time of the system for *μ* = 3.58 and set the random initial states just within individual chaotic bands instead of interval [0, 1]. The result shows that the synchronization times decay monotonically as the coupling strength increases (see Fig. 12(a)), except the tiny peak at *ε* = 0.405 in the case where the initial states are selected in band-1. In addition, we set the control parameter of the single maps to *μ* = 3.88 where the chaotic attractor has only one band in phase space, and estimate the corresponding synchronization times at different tolerances. The results are plotted in Fig. 12(b). It is found that the synchronization times decay non-monotonically as *ε* increases, except the case of Δ*R* = 10^−4^ where there is an indistinct peak around *ε* = 0.765 (here, the threshold of the coupling strength for the stable synchronous orbit is *ε*_m_ = 0.6500). In these cases, there is only one band and no competition among attractions from different periodic points or chaotic bands. Therefore, the two exceptions occurred in Fig. 12(a,b) are only due to the interplay between the local dynamics and the coupling term. This answers the question why the non-monotonic coupling strength dependence of the synchronization time was seldom reported since in most of the previous works there is only one chaotic band in the phase space of the single map.

**Figure 13:**
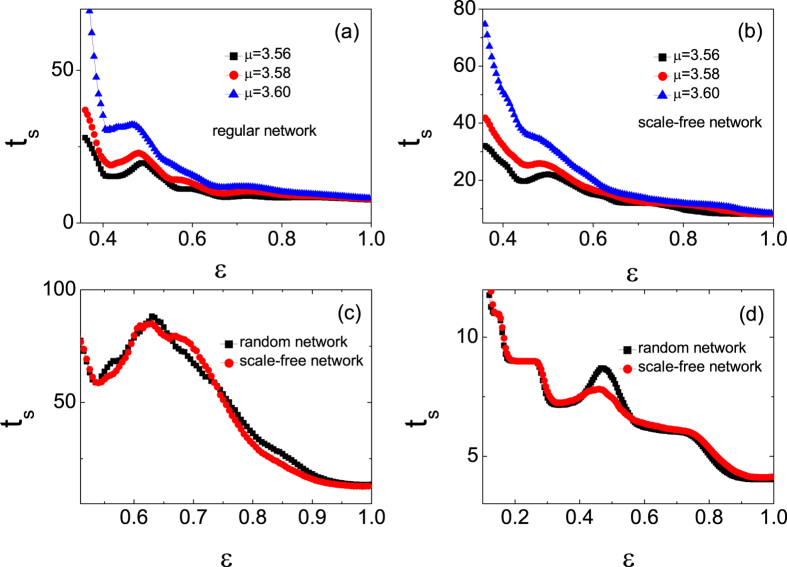
The synchronization times versus coupling strength *ε*. (**a**) Logistic maps are coupled via regular networks. The average degree is 640. (**b**) Logistic maps are coupled via scale-free networks. The average degree is 〈*k*〉 = 20. (**c**) The DIPWLMs are coupled by the random network (squares) and scale-free networks (circles). The average degree is 20. The 2-bands chaotic states (*μ* = 0.062) are used in simulations. (**d**) The TPLMGs are coupled by the random network (squares) and scale-free networks (circles) with the average degree 〈*k*〉 = 20. The 2-periodic states (*v*_*c*_ = 110) are used in simulations. The network size is *N* = 1000. The results are averaged over 10000 realizations. The threshold of synchronization is Δ*R*_*t*_ < 10^−8^.

The results presented previously suggest that the synchronous slowing down is induced by the competition between the local dynamics and the common evolution, where the multiple period or multiple band structure of maps plays a key role in the appearing of the phenomenon. To verify the generality of the mechanism, we have also studied other types of network structures and maps as shown in Fig. 13. For regular lattice of coupled logistic maps, synchronization is difficult. When the connection radius is large enough so that synchrony can be achieved, the non-monotonic variance of synchrony speed with respect to the coupling strength is also obtained (Fig. 13(a)). The same phenomenon is found in the coupled logistic maps with the same parameters by scale-free topologies (see Fig. 13(b)).

Besides the logistic map, the coupled systems of both discontinuous and invertible piece-wise linear maps (DIPWLM)^42^ and the coupled two-piece linear maps with a gap (TPLMG) are also studied. The DIPWLM is a simplified model for a relaxation oscillator^43^, which can describe many realistic systems including cardiopathy, relaxation and impact oscillators, relay control systems, and DC-DC converters. It is described by the following equations:


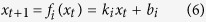


where *i* = 1, 2, 3, 4 and


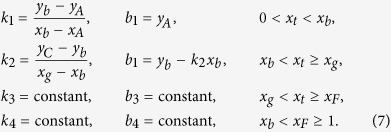


with *y*_*b*_ = *b*_1_ − *μ*, where *μ* is selected as the control parameter. The values of the parameters are selected as *y*_*A*_ = 0.0203921, *y*_*C*_ = 0.46, *y*_*G*_ = *y*_*A*_, *x*_*b*_ = 0.107663, *x*_*g*_ = 0.35, *x*_*F*_ = 0.497121, *k*_3_ = 3.07055, *b*_3_ = −0.530165, *k*_4_ = 0.405507, and *b*_4_ = −0.201586. The TPLMG was used to describe the period-adding bifurcation scenario of rat’s neuron^41^. The dynamics is like


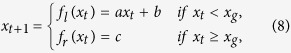


where *a* and *b* are the slope and intercept of the left map, and *c* stands for a constant. *a* = 3.83 × 10^−4^ *μ* + 0.9749, *b* = 5.628 × 10^−5^ *μ* + 0.0054, *x*_*g*_ = −6.3865 × 10^−5^ *μ* + 0.2198, and *c* = 1.38 × 10^−4^ *μ* + 0.1962. Here, *μ* is the control parameter. In the coupled systems of these two maps, we also find the synchronous slowing down. Figure 13(c,d) show the results of these coupled maps on random networks and scale-free networks, respectively, where DIPWLM is in the 2-bands chaotic state and TPLMG is in the 2-period state. For the control parameters selected in the calculations, a common feature of the above mentioned local maps is that their attractors show multiple-period or multiple-band structures. Therefore, we may conclude that the phenomenon observed in the coupled logistic maps is generic for the coupled systems consisting of maps whose attractors show multiple-period or multiple-band structures.

In the previous discussion, we have verified that the synchronous slowing down is generic in coupled maps. Another question we concern about is whether the same phenomenon can exist in continuous time systems. We present two examples of low-dimensional continuous time systems. The first example is the coupled Kuramoto models. Its dynamics is described by,


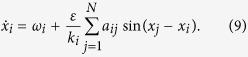


**Figure 14:**
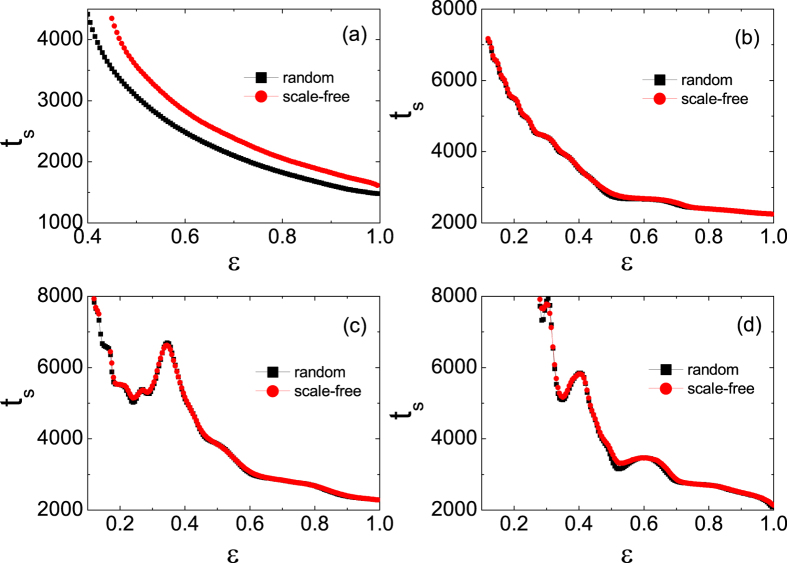
(**a**) The synchronization time of coupled Kuramoto models versus coupling strength *ε*. (**b**–**d**) The synchronization time of coupled Duffing oscillators versus coupling strength *ε*. The Duffing oscillators are in the 1-period states (*F* = 0.26), the 2-period states (*F* = 0.28), and the 4-period states (*F* = 0.29), respectively. The random network (squares) and scale-free networks (circles) are used. The average degree of networks is 20. The network size is *N* = 1000. The results are averaged over 2000 realizations. The threshold of synchronization is Δ*R*_*t*_ < 10^−8^.

Identical with the result of the coupled logistic maps shown in Fig. 12(b), the synchronization time in this system decreases monotonically with the increase of coupling strength (see Fig. 14 (a)). The reason is that the single Kuramoto model has only period-1 trajectory in phase space. While in the networks of coupled Duffing oscillators, different scenes are observed. The Duffing oscillator reads,


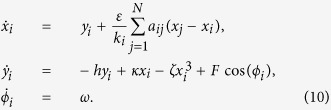


Here the parameters are *h* = 0.3, *κ* = 1, and *ς* = 1. With these values of parameters, the single Duffing oscillator exhibits the period-doubling bifurcations as

*F* increases. We simulate the coupled Duffing oscillators in 1-period (*F* = 0.26), 2-period (*F* = 0.28) and 4-period states (*F* = 0.29). The variations of the synchronization time with respect to the coupling strength are shown in Fig. 14(b–d), respectively. Here, the monotonic behavior of the synchronization time is observed again for the 1-period state of the oscillators, but the non-monotonic ones are displayed for the multi-period cases. This example suggests that the slowing down of synchrony may also exist in the coupled continuous-time systems when they are in the multi-period state.

## Discussion

In summary, we have studied the synchronization process of coupled logistic maps. It is unveiled that the synchronization time non-monotonically changes with the coupling strength in the networks of maps that are in the multiple periodic or multiple bands chaotic states. We also present the mechanism of this phenomenon. The result shows that there are two typical kinds of transient process before the system reaches complete synchronization. The synchronization processes are very different in weak and strong couplings. When the coupling is weak, the node states are attracted to the stable orbits or chaotic attractors of the maps and evolves toward the synchronized orbit almost individually, i.e., in a less coherent way. When the coupling is strong, the collective dynamics dominates the evolution of the system. The node states evolve as a whole, or in a high coherent way, toward the stable orbit on the synchronized manifold. In a mediate coupling strength, the interplay between the two paths is responsible for the slowing down of the synchronization. The competition among attractions from different periodic points of a multiple-period cycle or different chaotic bands of a multiple-band attractor enhances this slowing down. This behavior is a novel phenomenon, which is different from the critical slowing down at the boundary of the synchronization region^33^. The existence of different synchronization paths is also proven by the finite-time Lyapunov exponent and its distribution.

The results in this work can shed light on the effect of phase space structure on the synchronization process. The new observations of different synchronization paths and the slowing down of the synchronization process at the mediate coupling strength may be useful for optimizing the synchronization process. It can also find applications in explaining and controlling the process of some practical systems and neuron systems. The possibility can be revealed by the following fact. In the functioning of olfactory discrimination, a group of neurons synchronize on a fast time scale (30–100 Hz), then neurons lock into a specific phase on an intermediate time scale (4–8 Hz). Olfactory representation could be elaborated across sniffing cycles (hundreds of milliseconds)^29^. In human visual system, visual processing can be achieved in under 150 ms^30^, and the processing is based on feed-forward propagation of synchronous spiking^44^. In these systems where synchrony speed is important, synchronization is achieved quickly. Slightly slowing down may impact the functioning. In our results, the synchronization speed is high and the slowing down in the coupled systems with their local dynamics in multi-period and multi-band states is significant. It would be an interesting problem on how the slowing down affects the function of realistic systems. The application of our results in real systems will be a different type of work for future investigation. In addition, we would like to point out that the results in the current work may suggest that the synchronous slowing down is generic in both the coupled map systems and the coupled low dimensional continuous time systems.

## Additional Information

**How to cite this article**: Wang, S.-J. *et al*. Synchronous slowing down in coupled logistic maps via random network topology. *Sci. Rep*. **6**, 23448; doi: 10.1038/srep23448 (2016).
